# Improved healing and tissue regeneration for prevention and treatment of pelvic floor disorders: from reactive treatment to proactive prevention

**DOI:** 10.3389/fbioe.2026.1895072

**Published:** 2026-08-24

**Authors:** Basak Akin, Sarah A. Sinclair, Jan Paul Roovers, Zeliha Guler

**Affiliations:** 1 Department of Obstetrics and Gynecology, Amsterdam UMC, Location AMC, University of Amsterdam, Amsterdam, Netherlands; 2 Amsterdam Reproduction and Development (AR&D) Research Institute, Amsterdam UMC, Amsterdam, Netherlands

**Keywords:** biomateials, pelvic floor disorders (PFDs), prevention, regeneration, tissue engineering, wound healing

## Abstract

Pelvic floor disorders (PFDs), including pelvic organ prolapse (POP), stress urinary incontinence (SUI), and obstetric fistula, impose a major global health burden. Largely driven by birth-related trauma, they reduce quality of life and can cause physical, psychological, and social consequences. Current surgical and conservative treatments often fail to address underlying tissue damage and impaired healing, contributing to high recurrence; up to 30% of patients require repeat procedures. Tissue engineering and regenerative medicine offer promising alternatives by targeting damaged pelvic tissues and promoting repair and regeneration. Enhanced healing strategies can potentially prevent PFD development by strengthening pelvic floor tissues during critical periods of vulnerability, such as pregnancy and childbirth, while also improving outcomes following injury or surgical intervention. This paradigm shift from reactive treatment to proactive prevention and biological restoration represents a transformative opportunity in PFD management. This review examines the current landscape of tissue regeneration strategies for PFDs. We explore emerging therapeutic approaches within the tissue engineering framework, including biofunctional materials, cell-based therapies, bioactive molecules, molecular interventions, and external stimulations designed to promote native tissue regeneration. This review provides an overview of impaired healing mechanisms in pelvic floor tissues and evaluate how targeted regenerative interventions address these deficiencies. We examine the clinical implications of regenerative strategies in PFD management, including reduced surgical burden, improved outcomes, and preventive interventions. Emphasis is placed on their translational potential from bench to bedside, to inform clinicians and researchers about innovative approaches that may fundamentally transform the prevention and treatment of pelvic floor disorders.

## 1 Introduction

Pelvic floor disorders (PFDs)—including pelvic organ prolapse, urinary and fecal incontinence, and related dysfunction—are highly prevalent conditions that arise from a mismatch between mechanical demands on the pelvic floor and the capacity of its connective and muscular tissues to maintain support and continence. Even though PFDs, pelvic organ prolapse (POP), stress urinary incontinence (SUI), and obstetric fistula are distinct conditions with different pathophysiology and therapeutic goals, yet they share childbirth-related mechanical injury and ECM remodeling. Beyond their clinical classification, PFDs are ultimately disorders of structure–function integrity: tissue injury, compromised extracellular matrix (ECM) homeostasis, neuromuscular impairment, and dysregulated wound repair converge to drive long-term functional decline (Alperin et al., 2022; Guler and Roovers, 2022; Ashton‐miller and DeLancey, 2007). Although these conditions are rarely life-threatening, their cumulative impact on quality of life, sexual health, participation in daily activities, and healthcare utilization is substantial.

From a healing and regeneration perspective, a central challenge in PFD management is that current therapies largely address symptoms or anatomical displacement rather than restoring durable tissue quality. Conservative approaches (e.g., pelvic floor muscle training, pessaries) can be effective for certain patients but do not directly rebuild compromised connective tissue architecture, fascia, ligaments, or muscle (van der Vaart et al., 2022). Surgical repair, while often necessary and can restore anatomy, relies on tissues that may already be biologically weakened and prone to maladaptive remodeling (Ameli et al., 2023; Coolen et al., 2018). In POP, recurrence and reoperation remain persistent concerns; similarly, SUI procedures can show declining long-term success (Alperin et al., 2022; Coolen et al., 2017; Araco et al., 2008). In addition, surgical implants used for correcting pelvic floor anatomy were associated with clinical complications (Abdel-Fattah et al., 2011; Ford et al., 2015; Abed et al., 2011; Barone et al., 2016; Brătilă et al., 2017; Chapple et al., 2017). These clinical patterns underscore that the pelvic floor is not merely a structure to be “tightened,” but a living, load-bearing system that must heal, remodel, and adapt over time.

These limitations have catalyzed a shift from purely reparative strategies toward approaches that actively modulate healing and promote regeneration. Notably, tissue engineering solutions initially entered the pelvic floor field with a strong emphasis on mechanical reinforcement—biomaterials designed to restore support by bearing load. Over time, however, it has become clear that mechanical support alone is insufficient, and that successful outcomes depend on constructive host integration, controlled inflammation, and restoration of functional ECM and muscle architecture (Guler and Roovers, 2022; Gong and Xia, 2019; Verhorstert K. W. et al., 2022). Consequently, contemporary tissue engineering for PFDs has increasingly focused on treatment through improved tissue regeneration: designing biomaterials and biologic adjuncts that guide cell behavior, orchestrate immunomodulation, and enable remodeling that approximates native pelvic tissue composition and mechanics.

Building on many years of translational tissue engineering research in pelvic floor health, together with insights from clinical (uro)gynecology studies, our findings support a key shift in perspective: regeneration is not only a “better repair,” but also a potential route toward earlier, more preventive intervention. Our studies suggest that impaired healing and matrix homeostasis are not peripheral details in pelvic floor disorder management, but major determinants of durable outcomes. As evidence accumulates that targeted modulation of healing can improve pelvic tissue regeneration—and in some contexts may achieve clinically meaningful restoration—the field is beginning to ask a broader, prevention-oriented question: can regenerative approaches be deployed earlier, before irreversible tissue failure and symptomatic disease occur? This perspective aligns with the biological reality that childbirth-related trauma, aging, and chronic mechanical loading initiate progressive remodeling trajectories long before patients present for surgical care (Andrews et al., 2006; Glazener et al., 2014). If regenerative interventions can be timed to intercept maladaptive repair, reinforce tissue resilience, and normalize remodeling, they may ultimately reduce incidence, delay progression, or lower recurrence risk following conservative or surgical management.

This mini review examines the healing deficit at the core of PFD pathophysiology and synthesizes tissue engineering strategies aimed at restoring pelvic floor tissue integrity. We highlight key biological processes that govern repair and failure, discuss design criteria for regenerative constructs in this mechanically demanding environment, and summarize emerging biomaterial- and biologic-based approaches. Finally, we consider the translational steps required to move from regenerative treatments toward prevention-focused strategies that preserve pelvic floor function across the life course.

## 2 Why pelvic floor tissues fail: injury, remodeling, and impaired healing

The pelvic floor is a coordinated support system composed of skeletal and smooth muscle, connective tissue, fascia, ligaments, vessels, and nerves. Its ability to maintain continence and organ support depends on intact structure–function relationships across these compartments (Alperin et al., 2022; Ashton‐miller and DeLancey, 2007; DeLancey et al., 2007a; DeLancey et al., 2007b).

Childbirth is the main source of pelvic floor injury because vaginal delivery exposes tissues to extreme stretch, compression, and ischemia. Levator ani defects on MRI are strongly associated with later stress urinary incontinence and pelvic organ prolapse, while perineal and vaginal lacerations can lead to pain, infection, anal sphincter injury, and long-term dysfunction (DeLancey et al., 2007a; DeLancey et al., 2003; Blomquist et al., 2018; Buchanan et al., 2023).

**FIGURE 1:**
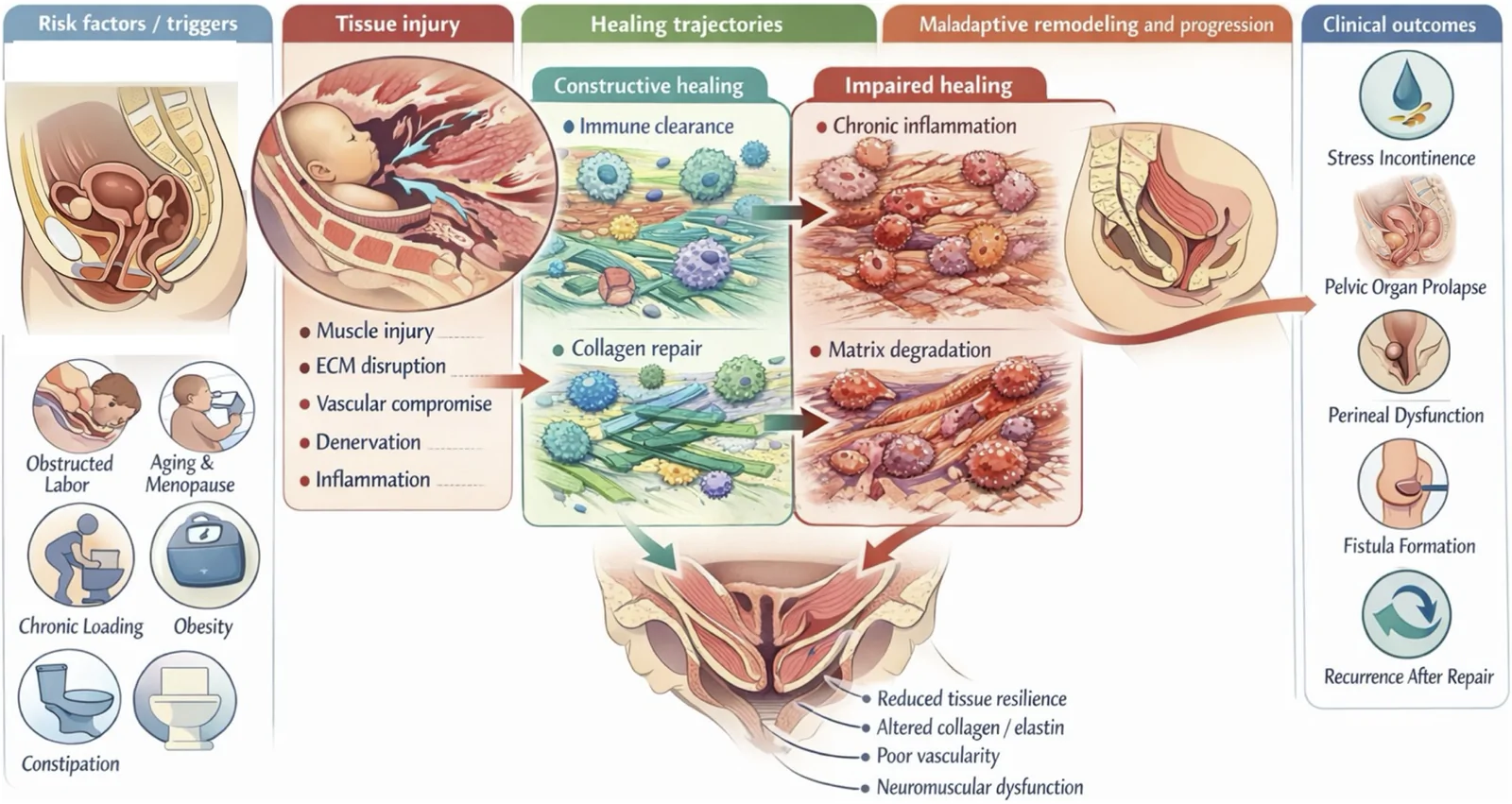
Pathophysiology and disease progression of pelvic floor disorders. Pelvic floor disorders arise from the interaction between injury-related triggers, impaired healing, and maladaptive remodeling. Childbirth trauma, obstructed labor, aging, menopause, and chronic mechanical loading can cause muscle, connective tissue, vascular, and nerve injury. When healing is dysregulated, persistent inflammation, extracellular matrix disruption, fibrosis, and mechanically weak repair may develop, ultimately contributing to stress urinary incontinence, pelvic organ prolapse, fistula formation, and recurrence after repair.

In obstructed labor, prolonged ischemia may cause tissue necrosis and fistula formation, and defective extracellular matrix remodeling can impair closure and healing (Adamowicz et al., 2019). Aging, menopause, and chronic mechanical loading further reduce vascularity, alter matrix turnover, impair regenerative capacity, and promote a low-grade inflammatory milieu that undermines repair (Guler and Roovers, 2022; Rieger et al., 2021; Tim and Mazur-Bialy, 2021). Although acute strain such as short-term weightlifting may be tolerated in healthy young women, repetitive high-pressure loading and constipation can still contribute to maladaptive remodeling over time (Figure 1) (Tim and Mazur-Bialy, 2021; Skaug et al., 2023).

Although POP, SUI, and obstetric fistula arise from distinct anatomical contexts and injury mechanisms, they share the common pathological feature of a disrupted extracellular matrix (ECM) homeostasis and impaired tissue repair. In POP, progressive weakening of the pelvic support tissues is associated with altered collagen composition, elastin fragmentation, dysregulated matrix remodeling, and reduced mechanical integrity (Kerkhof et al., 2009; Chen and Yeh, 2011). Similarly, SUI is characterized by degeneration of the periurethral support tissues, with decreased collagen and elastin content and impaired ECM remodeling that compromises urethral support and continence (Chen and Yeh, 2011). In obstetric fistula formation, prolonged ischemia during obstructed labor can lead to tissue necrosis followed by fibrotic healing and abnormal ECM architecture, excessive collagen deposition, persistent inflammation, can impair wound healing (Adamowicz et al., 2019; Satora et al., 2023; Shiferaw et al., 2026). Despite these distinct etiologies, all three conditions converge on a shared pathology of ECM imbalance and impaired remodeling sustained from vaginal delivery, highlighting restoration of a structurally organized, mechanically competent, and biologically functional ECM as a central goal of regenerative therapeutic strategies.

Regardless of cause, pelvic floor disorders develop when tissue repair is incomplete or maladaptive. Normal healing progresses through overlapping inflammatory, proliferative, and remodeling phases in which immune cells clear debris, fibroblasts deposit provisional matrix, and collagen III-rich tissue is reorganized toward stronger collagen I- and elastin-containing architecture. In pelvic tissues, this program is often disrupted by persistent loading, ischemia, hormonal change, oxidative stress, and dysregulated immunity, leading to excessive matrix degradation, abnormal myofibroblast activity, fibrosis, or mechanically weak tissue. Effective prevention and treatment therefore require not only symptom control, but improved tissue healing and avoidance of maladaptive remodeling trajectories (Guler and Roovers, 2022; Chi et al., 2022).

## 3 Current treatment and prevention strategies: what they do (and do not) fix

Conventional prevention strategies, including pelvic floor muscle training, lifestyle counseling, and pessaries, are usually introduced after early dysfunction appears and can reduce symptoms or slow progression. Pelvic floor muscle training during pregnancy can lower postpartum urinary incontinence short-term, and perineal massage, warm compresses, and selected protective devices may reduce perineal trauma (Johannessen et al., 2021; Stafne et al., 2022; Akbarzadeh et al., 2016; Rodrigues et al., 2023; André et al., 2024). However, these measures do not directly restore injured connective tissue or muscle, and long-term benefits appear limited, with no lasting effect shown at 12 years in follow-up studies (Glazener et al., 2014).

Lifestyle optimization remains important for tissue health but is rarely sufficient as a regenerative strategy on its own. Obesity increases intra-abdominal pressure and chronic inflammation, smoking impairs oxygenation and collagen synthesis, and nutrition supports repair, although evidence for specific supplements remains inconsistent (Fisher-Yosef et al., 2025) (Lababidi et al., 2023; Hill et al., 2025; Stanescu et al., 2025).

Surgery remains necessary for many patients with advanced disease, but recurrence and reoperation remain common after both prolapse and incontinence procedures. Permanent polypropylene mesh was introduced to improve durability, yet erosion, chronic pain, foreign body response, and poor integration limited its long-term safety and acceptability (Coolen et al., 2018; Coolen et al., 2017; Ford et al., 2015; Kowalik et al., 2019). These shortcomings highlight the need for therapies that provide support while also improving tissue quality and remodeling.

## 4 A regenerative framework: guiding healing instead of only correcting anatomy

**FIGURE 2:**
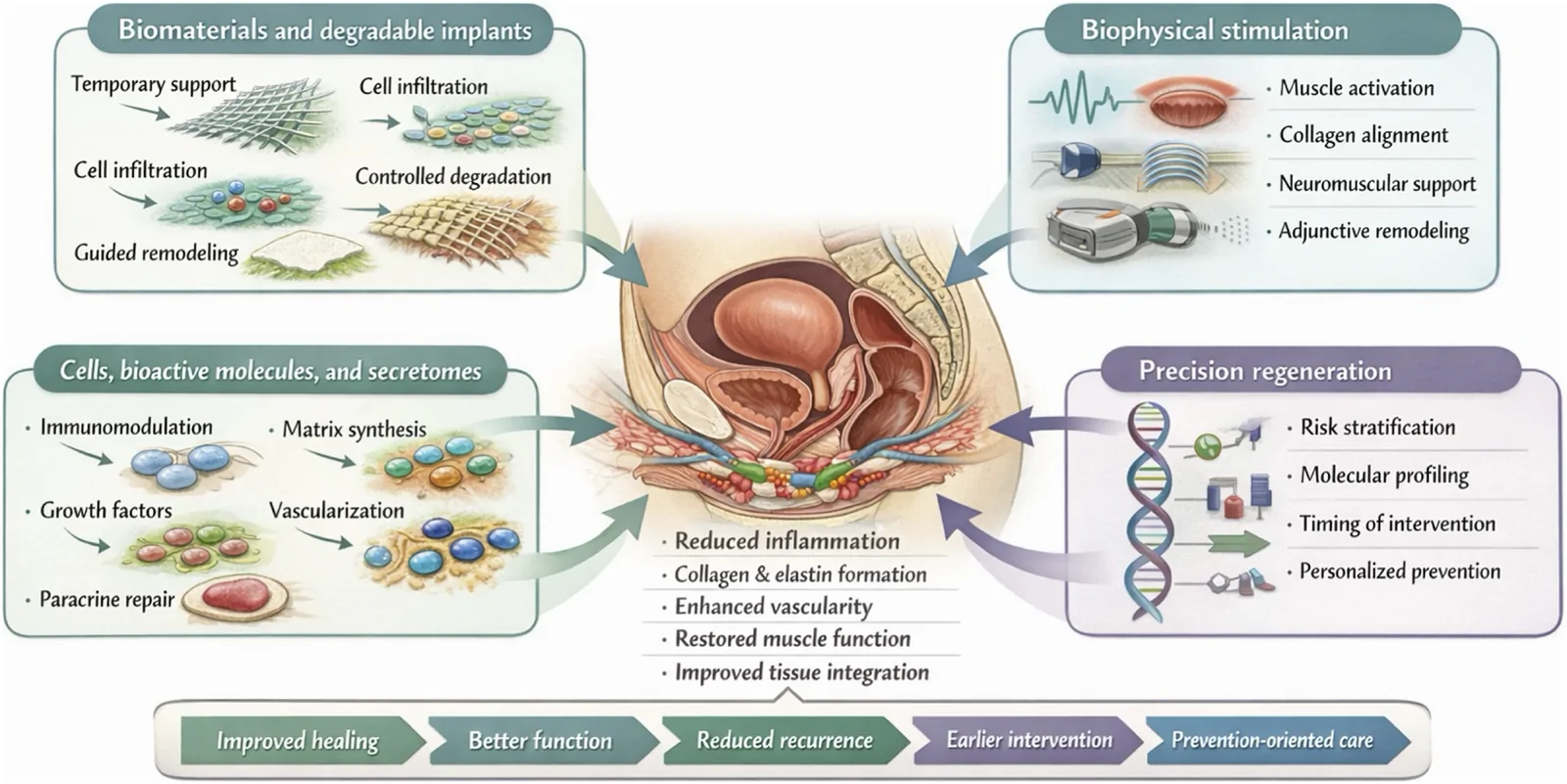
Regenerative strategies framework for pelvic floor disorders. Regenerative pelvic floor care combines biomaterials, biologic therapies, biophysical stimulation, and molecular stratification to guide constructive healing rather than passive structural repair alone. Degradable scaffolds and hydrogels can provide temporary support and instructive microenvironments, while cells, growth factors, platelet-rich plasma, and extracellular vesicles may promote immunomodulation, matrix synthesis, and vascularization. Biophysical stimulation can support neuromuscular recovery and remodeling, and precision approaches may help match interventions to tissue phenotype, biologic vulnerability, and timing of treatment. Together, these strategies aim to improve healing, restore function, reduce recurrence, and support earlier preventive intervention.

Because current prevention and treatment strategies do not reliably restore durable pelvic tissue quality, attention has shifted toward a regenerative framework that guides healing rather than only correcting anatomy. In pelvic floor tissue engineering, this framework centers on biomaterials, cells, or cell-derived signals, and external stimuli. Although different types of PFDs have distinct pathophysiologies, preventive regenerative strategies converge on promoting tissue repair and limiting maladaptive ECM remodeling before the development of persistent and established damage. Similarly, regenerative treatments aim to restore function by supporting effective healing and remodeling of tissues affected by mechanical strain. In this context, biomaterials provide structural and biochemical cues, cells and secretomes contribute regenerative and immunomodulatory activity, and physical stimulation can help direct remodeling toward functional recovery. Molecular and genetic insights add a precision layer by helping explain susceptibility, healing failure, and recurrence (Figure 2) (Guler and Roovers, 2022; Wu et al., 2020). Together, these approaches aim to create conditions for constructive remodeling in a mechanically demanding environment. Summary of all included studies are listed in Table 1.

**TABLE 1:** The overview of the clinical and preclinical studies included in this mini review, summarizing the investigated regenerative strategies, study models, target conditions, and key findings.

Overview of the included studies									
Type of study	Paper title and date published	Authors	Study subjects	Study design	PFD condition	Intervention and route of administration	Timing of administration& follow up	Outcome measures	Key findings (and safety)
Cells/Bioactive molecules/Cell free secretomes									
Clinical study	1-year follow-up of autologous muscle-derived stem cell injection pilot study to treat stress urinary incontinence (2008)	Carr, L.K., Steele, D., Steele, S. et al.	Humans	Case report	SUI	1-year follow up of 8 women in safety and feasibility trial Muscle derived stem cells (MDSC) injections MDSCs were injected subjects received up to four injections First three women: Transurethral in 3 o’clock+9 o’clock positions Second two women: Transurethral at 3, 6, 9, and 12 o’clock positions Last three wpmen: Periurethral injection at 3, 6, 9, and 12 o’clock positions Additionally, three of these patients received a reinjection Using the four target periurethral approach after the 3-month Follow-up visit	8 patients included Three of these patients received a reinjection Using the four target periurethral approach after the 3-month Follow-up visit Follow up: 1 + 3+6 + 12 months after injection	Pre + post injection pad weights, bladder diaries and quality of life measures	Improvement in SUI in remaining five women, with one subject achieving total continence that was maintained until study endpoint Onset of improvement is delayed following injection, suggesting that restoring muscle function may be the mechanism of action unlike standard bulking agents. Deeper delivery of MDSCs into the external sphincter appears to be important for successful outcomes Dose of cells has yet to be determined No serious adverse events
	Effect of autologous muscle-derived cells in the treatment of urinary incontinence in female patients with intrinsic sphincter deficiency and epispadias: A prospective study (2016)	Sharifiaghdas F, Tajalli F, Taheri M, Naji M, Moghadasali R, Aghdami N, Baharvand H, Azimian V, Jaroughi N.	Humans	Prospective study	SUI	Injection of autologous muscle derived cells (AMDC) Endoscopic or transurethral injection at 2,5,7,11, o’clock positions Follow up injections after 6 months in selected cases	10 included patients Follow up: 1 + 6+12 + 36 months	Cough stress test and 1-h pad test, incontinence impact questionnaire test Multichannel urodynamic Study and MUCP profilometry performed before and 12 months after last treatment	Three patients were cured, four patients improved, three did not improve Higher doses of AMSC showed significantly more efficacy compared to low doses Muscle-derived cell therapy might represent a minimally invasive and safe procedure in the treatment of patients with severe and complicated stress urinary incontinence There was no major adverse effect related to the treatment This study supports the safety of treatment
​	Autologous muscle-derived cell injection for treatment of female stress urinary incontinence: A single- arm clinical trial with 24-month follow-up (2019)	Sharifiaghdas F, Zohrabi F, Moghadasali R, Shekarchian S, Jaroughi N, Bolurieh T, Baharvand H, Aghdami N.	Humans	Clinical trial	SUI	Transurethral injection of autologous muscle derived cells (MDC)	20 included patients Follow up: 1 week+ 1 + 3+6 + 9+12 + 24 months	Physical examinations (cough stress tests), 1-h pad test, in-continence impact Questionnaire-7 (IIQ-7), and urogenital distress inventory (UDI-6) scoring Multichannel urodynamic study were performed before and 24 months after the intervention	MDC therapy was a minimally invasive and safe procedure for treatment of female patients with pure SUI. However, currently, the efficacy of this type of treatment for SUI is not sufficiently high and multi-center randomized clinical trials are required to be conducted before reaching a concrete conclusion The intervention produced no serious AE during the trial
	Autologous adipose stem cells in treatment of female stress urinary incontinence: Results of a pilot study (2014)	Kuismanen K, Sartoneva R, Haimi S, Mannerström B, Tomás E, Miettinen S, Nieminen K.	Humans	Pilot study	SUI	Transurethral injections of autologous adipose stem cells (ASCs) combined with bovine collagen gel and saline The injections were placed directly under mucosa: 1.5 cm distal from the urethral neck at 3o and 9 o’clock Two additional concomitant injections of ASCs mixed with saline solution performed more distally to bring the ASCs in contact with the urethral musculature	5 included patients Follow up: 3 + 6+ 12 months	Gynecological examination, vaginal ultrasonography, cough test, 24-h pad test, standardized questionnaires, and urodynamic evaluations (at 6 months), evaluations on quality of life	At 6 months: 1 of 5 patients displayed a negative cough test At 1 year: 3 of 5 patients had negative cough test and two of them were satisfied and did not wish further treatment for SUI. The treatment with autologous ASCs has proven safe and well tolerated. However, the feasibility and efficacy of the treatment were not optimal; therefore, additional research is needed There were no major complications after transurethral injections. The injection has proven safe and well tolerated
	Human cord blood stem cell therapy for treatment of stress urinary incontinence (2010)	Lee CN, Jang JB, Kim JY, Koh C, Baek JY, Lee KJ.	Humans	Prospective study	SUI	Transurethral cord blood stem cell injection Injection into the urethra Under cystoscope in the 4o and 8 o’clock positions Two injections per patients performed	39 included patients Follow up: 1 + 3+12 months	Patients’ voiding diaries, patient satisfaction Test A 3-month postoperative Urodynamic study	Continuous improvement over the year shown in the Patient’s satisfaction results. Intrinsic sphincter deficiency and mixed stress incontinency improved in the ten patients evaluated by urodynamic study The results suggest that transurethral umbilical cord blood stem cell injection is an effective treatment for women with all types of stress urinary incontinence No postoperative complications were observed
​	Autologous platelet rich plasma (A-PRP) combined with pelvic floor muscle training for the treatment of female stress urinary incontinence (SUI): A randomized control clinical trial (2024)	Saraluck A, Chinthakanan O, Kijmanawat A, Aimjirakul K, Wattanayingcharoenchai R, Manonai J	Humans	RCT	SUI	Comparison autologous platelet rich plasma (A-PRP) injection in combination with pelvic floor muscle training (PFMT)to PFMT alone, check for adverse effects Two injections of A-PRP were administered with a 1-month interval between injections in the A-PRP injection + PFMT group. Both groups received PFMT.	60 included patients 31 patients: A-PRP + PFMT group 29 patients: PFMT group Follow up: 1 + 3 months Follow up: 2 + 5 months	The primary outcome was determined using the 1-h pad weight test (PWT) Secondary outcomes were measured using the incontinence quality of life questionnaire, item 11 on the international consultation on incontinence questionnaire-female lower urinary tract symptoms questionnaire, patient global impression of improvement, and the percentage subjective improvement score	A-PRP + PFMT could be a treatment option for women with SUI. Large phase III randomized controlled trials are required to confirm findings There were no reports of adverse events following injection of A-PRP.
	A pilot study: Effectiveness of local injection of autologous platelet-rich plasma in treating women with stress urinary incontinence (2021)	Long, CY., Lin, KL., Shen, CR. et al.	Humans	Pilot study (prospective intervention study)	SUI	Autologous platelet rich plasma (A-PRP) injection into anterior wall where mid-urethra is located	20 included patients Follow up: 1 + 6 months follow up	Baseline and follow up: Self-reported questionnaires Secondary outcomes of sexual function and treatment effect sorted by age were analyzed with valid statistical methods	A-PRP is effective in relieving SUI symptoms at both 1 month and 6 months post-treatment It seems to have a trend that treatment success rate with cured and improved symptoms was slightly higher in the younger group, although it did not reach statistical significance (P = 0.07) Safety: No significant adverse reactions were reported
	The efficacy and mid-term durability of urethral sphincter injections of platelet-rich plasma in treatment of female stress urinary incontinence (2022)	Chiang CH, Kuo HC.	Humans	Prospective study	SUI (intrinsic sphincter deficiency)	Platelet-rich plasma (PRP) injection Injection to external sphincter at 5 sites with 4 treatments over monthly invervals	26 included patients Follow up: 12 months after fourth PRP treatment	Primary outcomes post treatment: Global response assessment (GRA, scored 0–3) score after four PRP treatments Secondary endpoints: Changes in visual analog scale (VAS) of SUI, urodynamic parameters	Repeated urethral sphincter injections of autologous PRP are a safe procedure that provides significant reduction in the severity of female SUI and a mid-term durability, suggesting PRP treatment is effective to increase urethral sphincter resistance for female SUI.
​	The use of platelet-rich plasma as a novel nonsurgical treatment of the female stress urinary incontinence: A prospective pilot study (2021)	Athanasiou S, Kalantzis C, Zacharakis D, Kathopoulis N, Pontikaki A, Grigoriadis T	Humans	Prospective pilot study	SUI	Platelet –rich plasma (PRP) injection Two PRP injections to lower one-third of anterior wall at 4–6 weeks intervals	20 included patients Follow up:1 + 3+6 months	Baseline: Urodynamic studies, 1-h pad test, international consultation on incontinence questionnaire–Female lower urinary tract symptoms, King’s health questionnaire Follow up (1,3,6 months): Baseline measurements and patient global impression scale of improvement	Platelet-rich plasma injections were both effective and safe at least in the short term and could be offered as an alternative outpatient procedure for the treatment of SUI. However, these encouraging findings warrant further investigation with randomized controlled trials Safety: No adverse effects were observed
	Platelet-rich plasma for the treatment of stress urinary Incontinence-A randomized trial (2024)	Grigoriadis T, Kalantzis C, Zacharakis D, Kathopoulis N, Prodromidou A, Xadzilia S, Athanasiou S.	Humans	RCT	SUI	Periurethral platelet –rich plasma (PRP) injection In PRP group Injections at 3 levels of urethra at 4–6 weeks intervals 2 PRP injections Sham injection: Sodium chloride 0.9%	50 included patients	Baseline: Urodynamic studies, 1-h pad test, international consultation on incontinence questionnaire—female lower urinary tract symptoms (ICIQ-FLUTS), patient global impression scale of improvement, King’s health questionnaire Follow up (1,3,6 months): 1-h pad test and completed the King’s health questionnaire and the ICIQ-FLUTS, primary outcome was the subjective evaluation as indicated by the response to question 11a of the ICIQ-FLUTS questionnaire	During follow-up, the mean score of the 11a question decreased significantly in the PRP group compared with sham. Subjective cure was significantly higher in the PRP group (32% vs. 4%, P < 0.001) A significant reduction of urine loss assessed on the 1-h pad test was observed in the PRP group compared with the sham group at 6-month follow-up. No adverse events were observed
Preclinical study	Combined treatment with CCR1-overexpressing mesenchymal stem cells and CCL7 enhances engraftment and promotes the recovery of simulated birth injury-induced stress urinary incontinence in rats (2020)	Jiang, H.-H., et al.	Rat (sprague–Dawley)	Preclinical experimental study	SUI	Intravenous injection of CCR1-overexpressing MSCs and local urethral injection of CCL7	Immediately after dual injury; 1 week follow-up	MSC engraftment in urethra, EUS EMG, pudendal nerve motor branch potentials (PNMBP), and leak point pressure (LPP)	Combined treatment increased MSC homing to injured tissues and improved functional recovery (higher LPP and EMG activity) compared to other groups
	IGF-1 regulates the growth of fibroblasts and extracellular matrix deposition in pelvic organ prolapse. Open medicine, 2020.15 (1): p. 833–840	Yin, Y., et al.	Human (vaginal fibroblasts)	*In vitro* cell culture and tissue analysis	POP	Transfection of human vaginal fibroblasts (HVFs) with IGF-1 siRNA (knockdown) or IGF-1 plasmid (overexpression)	N/A	Cell viability (MTT), apoptosis (flow cytometry), collagen I and III expression, MAPK and NF-κB signaling pathway markers	IGF-1 expression is reduced in POP tissues; knockdown of IGF-1 inhibited viability and decreased collagen production, while IGF-1 overexpression suppressed cell apoptosis, promoted fibroblast growth and collagen metabolism
	Secretomes of human pluripotent stem cell-derived smooth muscle cell progenitors upregulate extracellular matrix metabolism in the lower urinary tract and vagina (2021)	Zhuang, G., et al.	Rat and human (vaginal fibroblasts)	Preclinical experimental study (*in vitro* and *in vivo*)	SUI	Peri-urethral injection of concentrated conditioned medium (CM) from human pluripotent stem cell-derived smooth muscle progenitor cells	Three weeks after injury; once weekly for 3 weeks; 5-week follow-up	Leak point pressure (LPP), ECM gene expression (collagen I, III, elastin), protein expression, and smoothelin immunofluorescence (myogenic response)	CM treatment significantly improved urethral function (LPP) in SUI rats and upregulated structural ECM components
​	BMSCs-EVs alleviate pelvic floor dysfunction in mice by reducing inflammation and promoting tissue regeneration. In vivo, 2024.38 (6): p 2680–2687	Hu, L. and C. Chen	Mouse	Preclinical experimental study	SUI/POP	Tail vein injection of BMSC-derived extracellular vesicles (EVs)	Once every 3 days for 2 weeks following simulated injury	Histopathology (HE, masson, sirius red), immunohistochemistry (Ki-67, LGR7), and protein levels (elastin, FBLN5)	BMSC-EVs alleviated pelvic floor dysfunction by reducing inflammation and fibrosis, promoting tissue regeneration, and increased elastin and FBLN5 protein expression
Biomaterials and degradable implants, injectable gels									
Clinical study	Tension-free vaginal tape versus polyacrylamide hydrogel bulking agent for stress urinary incontinence: Patient choice and outcomes in Finland (2025)	Särkilahti L, Isaksson C, Mikkola TS.	Humans	RCT	SUI	Polyacrylamide hydrogel (PAHG) injection is offered as an alternative to tension-free vaginal tape (TVT) PAHG injection: Under endoscopic control at 1.5 cm from the vesicourethral junction, hydrogel was injected at the locations “10, 2, 5, and 7 o’clock	391 included patients Re-treatments and complications were collected at 2-year follow-up after each primary procedure	Cytoscopy 70° optic during operation Cough test	Similar clinical profiles in both TVT and PAHG groups suggest no specific demographic factors predict decision making After a 2-year follow-up, the overall complication rates were similar, with PAHG associated with a higher likelihood of requiring re-treatment, whereas TVT carried a greater risk of severe complications. The re-treatment rates were lower than previously reported, indicating that actual patients are fairly satisfied with their primary choice
​	Tension-free vaginal tape and polyacrylamide hydrogel injection for primary stress urinary incontinence: 3-Year followup from a randomized clinical trial (2022)	Itkonen Freitas AM, Isaksson C, Rahkola-Soisalo P, Tulokas S, Mentula M, Mikkola TS.	Humans	RCT follow up	SUI	Non-inferiority determination of polyacrylamide (PAHG) to tension-free vaginal tape (TVT)	223 included patients 110 patients: TVT group 113 patients: PAHG group This is the 3 year follow up	Primary outcome: Patient satisfaction, non-inferiority margin for the difference was 20% Secondary outcomes: Effectiveness and complications	In midterm followup, PAHG did not reach in patient satisfaction the noninferiority set in our study. Furthermore, mid urethral TVT slings show better subjective and objective cure rates than PAHG. However, complications were more often associated with TVT. Since the majority of PAHG treated women were also cured or improved, primary SUI women can be offered PAHG as a safe and durable alternative treatment
	Quality of life and sexual function after TVT surgery versus bulkamid injection for primary stress urinary incontinence: 1 year results from a randomized clinical trial (2021)	Itkonen Freitas AM, Mikkola TS, Rahkola-Soisalo P, Tulokas S, Mentula M	Humans	RCT	SUI	Comparison tension-free vaginal tape (TVT) and polyacrylamide hydrogel (PAHG) injection	224 included patients 111 patients: TVT group 113 patients: PAHG group 1 year follow up	Urogenital distress inventory (UDI-6), incontinence impact questionnaire, short form (IIQ-7), pelvic organ prolapse/Urinary incontinence sexual questionnaire (PISQ-12), RAND-36 item health survey (RAND-36)	In primary SUI, TVT and PAHG treatments both improved QoL and sexual function at 1 year. However, incontinence and health-related QoL scores were better in the TVT group. More pain compared to the baseline was reported after TVT, although there was no difference between groups. Clinical significance needs to be evaluated in long-term follow-up
	Seven-year efficacy and safety outcomes of bulkamid for the treatment of stress urinary incontinence (2021)	Brosche T, Kuhn A, Lobodasch K, Sokol ER.	Humans	Single center retrospective study	SUI	This is the 7-year follow up after bulkamid injection	388 included patients This is the 7 years follow up	Primary endpoint: Patient satisfaction measured on a four-point scale as cured, improved, unchanged, or worse Secondary outcomes: The number of incontinence pads used, international consultation on incontinence questionnaire-short form (ICIQ-UI SF) scores, visual analog scale quality of life (VAS QoL), reinjection rates, perioperative and postoperative complications	Bulkamid injections are an effective and safe first-line treatment option for women with SUI or stress-predominant MUI providing durable outcomes at 7 years
Preclinical study	Absorbable electrospun poly-4-hydroxybutyrate scaffolds as a potential solution for pelvic organ prolapse surgery (2022)	Verhorstert, K., et al.	Human (vaginal fibroblasts)	*In vitro* cell culture study	POP	Culture of vaginal fibroblasts on electrospun (ES) P4HB (0%, 1%, 2%, 5% E2) and knitted P4HB scaffolds	28-day culture period	Cell proliferation (alamar blue), ECM deposition (picrosirius red), gene expression (COL1A1, COL3A1, ELN, MMP-2, MMP-9, α-SMA), MMP-2 activity	Compared to knitted scaffolds, ES P4HB scaffolds improved cell response (proliferation and matrix deposition); a high collagen-i/III ratio indicates improved remodeling; and E2 loading was effective and sustained
	Evaluation of the short‐term host response and biomechanics of an absorbable poly‐4‐hydroxybutyrate scaffold in a sheep model following vaginal implantation (2022)	Diedrich, C.M., et al.	Sheep	Preclinical short-term experimental study	POP	Rectovaginal implantation of P4HB scaffolds fixed with non-degradable sutures	Follow-up at 60 and 180 days	Anatomical exam, biomechanical properties (stiffness, contractility), *in vivo* degradation (mw, SEM), histomorphology (H&E, MT, IHC for CD45 and α-SMA)	P4HB showed good mechanical support and resulted in moderate host response *in vivo*
	Characterisation of polycaprolactone scaffolds made by melt electrospinning writing for pelvic organ prolapse correction-a pilot study (2022)	Rynkevic, R., et al.	*In vitro* (comparison to sheep tissue)	*In vitro* pilot study	POP	3D printed polycaprolactone meshes (5 configurations); sterilization and degradation in neutral (PBS) and acidic (KHP) solutions	Follow-up up to 180 days	Fiber weight loss (degradation), mechanical behavior (uniaxial tensile testing), pore deformation analysis (SEM, ImageJ)	PCL meshes showed stiffness closer to the vaginal tissue of sheep and exhibited a more isotropic behavior compared to the non-degradable polypropylene restorelle mesh
​	Two-year preclinical evaluation of long-term absorbable poly-4-hydroxybutyrate scaffold for surgical correction of pelvic organ prolapse (2024)	Guler, Z., et al.	Sheep	Preclinical randomized trial	POP	Rectovaginal implantation; P4HB or PP mesh	12- And 24-month follow-up	Passive biomechanical properties (stiffness, ultimate tensile strength), *in vivo* degradation, histomorphology, collagen and elastin content	P4HB completely absorbed in 24 months; collagen amount increased compared to PP mesh. P4HB maintained tissue stiffness
	Evaluation of electrospun Poly‐4‐Hydroxybutyrate as biofunctional and degradable scaffold for pelvic organ prolapse in a vaginal sheep model (2025)	van Rest, K.L., et al.	Sheep	Preclinical experimental study	POP	Vaginal implantation; electrospun (ES) P4HB or ES P4HB-E2 (with estrogen) scaffolds	3 months follow-up	*In vivo* degradation, infection, biomechanics (stiffness/UTS), histomorphology	Scaffolds improved tissue biomechanics; ∼50% degradation seen in 3 months; adding estrogen did not create a statistical difference
	Fully absorbable poly-4-hydroxybutyrate implants exhibit more favorable cell-matrix interactions than polypropylene (2020)	Diedrich, C.M., et al.	Human (vaginal fibroblasts)	*In vitro* cell culture	POP	P4HB knit scaffolds (4 designs) vs. PP	28-day culture period	Cell attachment, proliferation, collagen deposition, knit mechanical properties	P4HB provided improved cell proliferation and matrix deposition compared to PP mesh; “diamond” design gave the most optimal results
​	A new method to repair recto-vaginal fistula: Use of human amniotic membrane in an animal model (2014)	Roshanravan, R., et al.	Dog	Prospective animal study	POP	Iatrogenic rectovaginal fistula repair; standard repair vs. HAM patch	6 weeks follow-up	Gross healing (infection/discharge), microscopic scoring (epithelialization, collagenization, necrosis)	HAM patch repair resulted in a higher healing score compared to standard repair
	Mechanical reinforcement of amniotic membranes for vesicovaginal fistula repair (2023)	Maljaars, L.P., et al.	Human (amniotic membrane (AM))	*In vitro* mechanical test study	Fistula	Single/multilayer HAM; P4HB or bovine pericardium reinforcement	4 and 8 weeks follow-up	Suture retention strength, uniaxial tensile test (tensile strength, elongation, modulus)	Single layer AM is fragile; P4HB reinforcement increased suture retention strength above surgical threshold
	The regenerative capacity of tissue-engineered amniotic membranes (2024)	Maljaars, L., et al	Human (vaginal Fibroblasts/AM)	*In vitro* regenerative response study	Fistula	Culture of vaginal fibroblasts on AM, crAM (cross-linked), P4HB-reinforced structures	28 days culture period	Proliferation, cell morphology, collagen deposition, MMP-2 activity (zymography)	Un-crosslinked AM provided best proliferation; P4HB-reinforced AM structures are functional and suitable for vesicovaginal fistula repair
	Collagen type I–loaded methacrylamide hyaluronic acid hydrogel microneedles alleviate stress urinary incontinence in mice: A novel treatment and prevention strategy (2023)	Zhang, S., et al.	Mouse	*In vitro* and *in vivo* experimental study	SUI	HAMA-COLI (collagen type I loaded) microneedles (MN); anterior vaginal wall injection	Immediately after injury; 14 days follow-up	Biocompatibility, urodynamic indices (BLPP), collagen metabolism, inflammation (IL-6, IL-10)	Microneedles successfully penetrated mucosa; improved BLPP values; increased collagen amount
​	Extracellular vesicles from Adipose‐Derived mesenchymal stem cells combined with PEG hydrogel alleviate maternal simulated birth injury in a rat model (2025)	Wang, Q., et al.	Rats	Preclinical experimental study	SUI/POP	PEG@EVs (stem cell-derived extracellular vesicles) hydrogel injection; simulated birth injury	3,5,7,14,21, and 28 days follow-up	Oxidative stress, ferroptosis markers (Nrf2, GPX4), inflammation, collagen deposition	PEG@EVs reduced oxidative damage and ferroptosis; triggered M1 to M2 macrophage conversion; accelerated collagen deposition and vaginal repair
	Vaginal delivery of tissue engineered endometrial mesenchymal stem/stromal cells in an aloe vera-alginate hydrogel alleviates maternal simulated birth injury (2021)	Paul, K., et al.	Rat and human (eMSCs)	Preclinical experimental study	SUI/POP	eMSCs with aloe vera-alginate hydrogel (Hyd/eMSC) injection	Injection after simulated birth injury; 1 and 6 weeks follow-up	Vaginal diameter, histomorphology (rugae), nanoscopic collagen analysis with AFM, macrophage response	Hydrogel/eMSC intervention restored vaginal wall integrity; prevented smooth muscle loss and corrected nanoscopic collagen structure
	Polyisocyanides as a substrate to trigger vaginal fibroblast functioning in an *in vitro* model for prolapse repair (2022)	Gudde, A.N., et al.	Human (fibroblast)	*In vitro* model study	POP	Culture of vaginal fibroblasts in PIC hydrogels with varying RGD peptide density	28-day culture period	Morphology, proliferation, ECM production (collagen/elastin), active MMP-2 secretion	High RGD density (181 microM) significantly increased cell spreading, proliferation, and collagen/elastin production
​	Injectable polyisocyanide hydrogel as healing supplement for connective tissue regeneration in an abdominal wound model (2023)	Gudde, A.N., et al.	Rabbit	Preclinical experimental study	POP	Abdominal wound model; injection of PIC hydrogel loaded with bFGF, E2, or ADSC	Immediately after injury; 14 and 42 days	Gross morphology, tissue stiffness (Young’s modulus), collagen/elastin, host response	All PIC formulations increased tissue stiffness and collagen over time; bioactive factors did not hinder healing; estrogen reduced PMNC infiltration
	Vaginal fibroblast behavior as a function of stiffness changes in a polyisocyanide hydrogel for prolapse repair (2023)	Gudde, A.N., et al.	Human (vaginal fibroblasts)	*In vitro* biomechanical model	POP	Culture of vaginal fibroblasts in PIC hydrogels with varying stiffness	28-day culture period	Effective Young’s modulus of the gel, cell proliferation, collagen quantification, gel contraction	Substrate stiffness directly affects cell behavior; highest collagen production seen in medium stiffness (0.4%) gel
	Growth factor immobilization to synthetic hydrogels: Bioactive bFGF‐Functionalized polyisocyanide hydrogels (2023)	van Velthoven, M.J., et al.	Human (ASC)	*In vitro* bioactivity study	POP	Covalent binding of bFGF to PIC hydrogel (PIC-bFGF	28-day culture period	Cell proliferation, viability (Live/Dead), collagen/elastin amount, gene expression (COL1A1, ELN)	PIC-bFGF remained bioactive for 28 days and did not create any difference in collagen levels at any concentration
​	Potential of estradiol‐functionalized polyisocyanide hydrogels for stimulating tissue regeneration of the pelvic floor (2024)	van Velthoven, M.J., et al.	Human (ASC/Cell lines)	*In vitro* bioactivity study	POP	Covalent binding of estradiol (E2) to PIC hydrogel (PIC-E2)	Varying culture periods	dsDNA (proliferation), target gene expression (TFF1, CCN2, ECM genes)	Covalently bound E2 remained bioactive and increased ECM gene expression (COL1A1, ELN) in ASCs; no cytotoxicity seen
Biophysical stimulation									
Clinical study	Clinical effect of electrical stimulation biofeedback therapy combined with pelvic floor functional exercise on postpartum pelvic organ prolapse (2021)	Zhong F, Miao W, Yu Z, Hong L, Deng N.	Humans	Prospective study	POP	Electrical stimulation biofeedback therapy and pelvic floor exercises or exercises alone	104 included patients 52 patients: Control group pelvic floor exercises 52 patients: Electrical stimulation biofeedback therapy combined with pelvic floor exercises	The clinical efficacy, pelvic floor pressure (contraction pressure, resting pressure, contraction duration), improvement of pelvic floor prolapse, pelvic floor surface muscle potential, quality of sex life and quality of life (PFIQ-7 score and PFDI-20 score)	Electrical stimulation biofeedback therapy combined with pelvic floor functional exercise has a noticeable curative effect and can significantly alleviate pelvic floor prolapse and improve the sex life and quality of life of patients
	The effect of electrical stimulation therapy with pelvic floor muscle exercise on stress urinary incontinence in middle-aged women: A nonequivalent comparison cohort study (2021)	Lim H, Kang JA, Park H	Humans	Nonequivalent comparison cohort study	SUI	Electrical stimulation therapy with pelvic floor muscle exercise (PFME) or PFME alone Participants in combination group: 3 times daily PFMEs under weekly telephone coaching, Electrical stimulation device twice daily for 8 weeks PFME group: Exercise alone without telephone coaching	54 included patients 27 patients: Self-exercises on pelvic floor group 27 patients: Combination group Follow up after 8 weeks	Lower urinary tract symptoms, urinary incontinence–related quality of life, bristol female lower urinary tract symptom instrument (BFLUTS), King’s health questionnaire (KHQ), a perineometer	Combined electrical stimulation and pelvic floor exercise was more effective in reducing severity of lower urinary tract symptoms, enhancing health-related quality of life, and increasing PMC pressure in middle-aged women with stress urinary incontinence than PFMEs alone
​	The effect of rehabilitation exercises combined with direct vagina low voltage low frequency electric stimulation on pelvic nerve electrophysiology and tissue function in primiparous women: A randomised controlled trial (2017)	Yang S, Sang W, Feng J, Zhao H, Li X, Li P, Fan H, Tang Z, Gao L	Humans	RCT	Developing PFD	Comparison rehabilitation exercise combined with direct vagina low voltage low frequency electric stimulation or only exercise on electrophysiology and tissue function after delivery Low frequency electrical stimulation performed 15 times: 3x week for 30 min beginning 6 weeks postpartum with rehabilitation exercises Rehabilitation exercises were kegel exercises and pelvic movements from 2 days to 3 months postpartum	189 included patients 63 patients: Postpartum training group 66 patients: Combination group 60 patients: Control group with postpartum guidance Followed for 3 months after delivery	POP-Q score assessment, incontinence severity score, pad test, modified oxford scale, pubic symphysis clearance, pelvic floor muscle electrophysiology	Rehabilitation exercises can promote healing of the maternal pubic symphysis and recovery of the pelvis The total electrical value, type I muscle fibre strength and type II muscle fibre strength were significantly increased in the combination group after treatment than before treatment Rehabilitation exercises combined with low frequency electrical stimulation were beneficial to the recovery of postpartum pelvic nerve tissue function, and a synergistic effect was observed when the two methods were combined

### 4.1 Cells, bioactive molecules, and cell-free secretomes

Cell-based therapies seek to repair pelvic tissues by delivering regenerative cells or harnessing their paracrine effects. Mesenchymal stromal cells, adipose-derived stem cells, bone marrow-derived cells, muscle-derived stem cells, and endometrial stromal cells have been studied mainly in stress urinary incontinence and, to a lesser extent, prolapse repair (Carr et al., 2008; Sharifiaghdas et al., 2016; Sharifiaghdas et al., 2019). In preclinical models, these approaches can improve elastin and collagen deposition, vascularization, inflammation control, and functional recovery after birth injury (Wu et al., 2011; Lin et al., 2010). Early clinical studies suggest that cell injection is feasible and generally safe, but efficacy remains variable, follow-up time is short, and heterogeneity in cell source and protocol limits firm conclusions (Carr et al., 2008; Kuismanen et al., 2014; Marta et al., 2025; Lee et al., 2010).

A major limitation of live-cell therapy is poor homing, retention, and persistence after delivery, prompting interest in strategies that improve localization and in cell-free approaches that capture paracrine benefit without requiring durable engraftment (Jiang et al., 2020). This has increased attention to platelet-rich plasma, growth factors, conditioned media, exosomes, and extracellular vesicles. Growth factors such as bFGF, CTGF, and IGF-1 can support fibroblast activity and matrix synthesis, while platelet-rich plasma offers a clinically accessible mixture of regenerative cues that has shown symptomatic improvement and good short-term safety in stress urinary incontinence studies, although evidence remains limited and durability is modest (Yin et al., 2020; Athanasiou et al., 2021; Grigoriadis et al., 2024; Chiang and Kuo, 2022; Verma et al., 2023; Long et al., 2021; van Velthoven et al., 2023). Extracellular vesicles are especially attractive because they can reduce inflammatory signaling, influence macrophage polarization, and promote matrix repair in preclinical models. Their translation is still constrained by variable preparation methods, short tissue persistence, limited delivery control, and small heterogeneous study cohorts, underscoring the need for larger studies and standardized protocols (Verma et al., 2023; Long et al., 2021; Zhuang et al., 2021; Hu and Chen, 2024; Itkonen Freitas et al., 2021; Itkonen Freitas et al., 2022).

### 4.2 Biomaterials and degradable implants

Biomaterials remain central to pelvic floor repair, but the field has moved away from inert reinforcement alone. This shift reflects the limitations of permanent polypropylene meshes, including mechanical mismatch, chronic foreign body response, fibrosis, erosion, and pain. Current efforts focus on bioactive, degradable, and mechanically compatible scaffolds that support cell infiltration, regulate inflammation, and guide remodeling toward functional tissue replacement (Wu et al., 2020; Verhorstert K. et al., 2022; Diedrich et al., 2022; Rynkevic et al., 2022; Guler et al., 2024; van Rest et al., 2025).

In our research group, we have approached PDFs as a wound healing challenge. Therefore, we have investigated different biomaterials that can trigger healing and regeneration of the pelvic floor around the window of surgery. We focused on the development of regenerative biomaterials for stress urinary incontinence, pelvic organ prolapse or vesicovaginal fistula repair, with the objective of creating materials that provide mechanical support while actively directing host tissue repair and long-term functional restoration. In addition, injectable hydrogels were explored as supplement bioactive biomaterials that can improve surgical success and reduce the recurrence of native tissue repair. The goal is to create materials that provide temporary support while directing host repair and long-term functional restoration, including injectable hydrogels that may enhance native tissue repair and reduce recurrence after surgery.

#### 4.2.1 Degradable implants and meshes

Permanent polypropylene meshes provided mechanical durability but were associated with erosions and chronic complications. Degradable scaffolds aim to provide early support while avoiding long-term foreign body burden and encouraging tissue replacement.

A prominent example is the development of fully absorbable poly-4-hydroxybutyrate (P4HB) meshes as alternatives to permanent polypropylene for prolapse repair. *In vitro*, P4HB supported more favorable vaginal fibroblast attachment and collagen deposition than polypropylene, and in a two-year ovine study it was associated with higher collagen content, no mesh exposure at 24 months, and a more favorable host response (Guler et al., 2024; Diedrich et al., 2020; Diedrich et al., 2022). Implant architecture and biofunctionalization, including estradiol-releasing constructs and electrospun extracellular matrix-like microstructures, also influence cell behavior and host integration, emphasizing that scaffold success depends on coordinated degradation, structure, mechanics, and biologic activity rather than degradability alone. Overall, successful implants must match pelvic tissue mechanics, limit chronic inflammation, degrade predictably, and support organized extracellular matrix deposition with durable integration (Guler and Roovers, 2022; Verhorstert et al., 2022b; van Rest et al., 2025). Overall, successful implants must match pelvic tissue mechanics, limit chronic inflammation, degrade predictably, and support organized extracellular matrix deposition with durable integration.

Vesicovaginal fistula repair also requires both mechanical support and constructive tissue regeneration. Human amniotic membrane is a collagen-rich, low-immunogenic biologic containing growth factors and immunomodulatory cues, and early animal and clinical studies suggest promise in urogenital fistula repair (Barski et al., 2015; Roshanravan et al., 2014). Hybrid scaffolds that reinforce amniotic membrane with P4HB aim to combine biologic signaling with improved structural performance for pelvic floor reconstruction (Maljaars et al., 2023; Maljaars et al., 2024).

#### 4.2.2 Injectable hydrogels

Hydrogels have long been used as bulking agents for stress urinary incontinence, but conventional bulking mainly offers temporary augmentation rather than regeneration (Brosche et al., 2021). Newer systems aim to create cell-instructive microenvironments for prevention or early intervention. Examples include collagen I-loaded hydrogel microneedles that improved urodynamics in mice, PEG hydrogels that sustained extracellular vesicle delivery and promoted collagen deposition and M2-like immunomodulation, and hydrogel systems that improved stem cell retention and differentiation (Zhang et al., 2023; Wang et al., 2025; Paul et al., 2021). Our group introduced an RGD-functionalized injectable polyisocyanide (PIC) hydrogel with tunable mechanical properties as a platform designed to combine injectability with biological guidance of host cells (Gudde et al., 2022; Gudde et al., 2023a; Gudde et al., 2023b). We subsequently demonstrated that PIC hydrogels support vaginal fibroblast proliferation and collagen deposition *in vitro*, while allowing stiffness-dependent modulation of cellular behavior, highlighting the importance of mechanical tuning in regenerative design (Gudde et al., 2023b). Building on this platform, we further incorporated bFGF and estradiol to better align the local biomaterial environment with the biological requirements of pelvic floor healing and tissue regeneration (van Velthoven et al., 2023; van Velthoven et al., 2024). These studies collectively illustrate that the future of injectable biomaterials in urogynecology lies not in transient volume augmentation alone, but in the development of microenvironments that can direct constructive remodeling in a controlled and biologically meaningful manner.

Clinical translation of biomaterials remains strongly shaped by the experience with polypropylene mesh. Although degradable and biofunctional scaffolds show encouraging preclinical results, wider adoption will require long-term evidence of safety, predictable degradation, and sustained clinical benefit in relevant models and trials.

### 4.3 Biophysical stimulation as a regenerative adjunct

External stimuli are the third major component of tissue engineering and include electrical stimulation, mechanotherapy, magnetic stimulation, radiofrequency, and other energy-based approaches (Baruch et al., 2025). These modalities may influence muscle activation, collagen alignment, vascularization, and matrix remodeling. Electrical stimulation is the most established clinically and can improve neuromuscular function, especially when combined with pelvic floor muscle training, while implantable stimulators have shown preclinical promise in reducing inflammation and restoring collagen metabolism (Zhong et al., 2021; Lim et al., 2021; Li et al., 2025; Min et al., 2017). However, mechanisms remain incompletely defined and long-term functional evidence is limited, so these approaches are best viewed as promising adjuncts rather than standalone regenerative solutions at present.

### 4.4 Toward precision regeneration: mmlecular and gene-informed strategies

Molecular and genetic insights are increasingly important for explaining who is at risk, why tissues fail, and how regenerative strategies might be tailored. Pelvic organ prolapse and stress urinary incontinence are linked to altered collagen and elastin homeostasis, matrix turnover, oxidative stress, inflammation, hormone-responsive pathways, and smooth muscle biology (Visco and Yuan, 2003; Hundley et al., 2008). Reported associations involve extracellular matrix and remodeling genes such as COL1A1, COL3A1, FBLN5, LOXL1, LAMC1, MMP-related pathways, and related regulators, suggesting that baseline biologic vulnerability interacts with childbirth trauma and aging to influence disease onset and recurrence (Ala et al., 2010; Isali et al., 2020; Chen et al., 2002; Jiang et al., 2024; Olafsdottir et al., 2020; Pujol-Gualdo et al., 2022). Although true gene therapy remains experimental, molecular profiling may already support risk stratification, phenotype-specific prevention, better timing of intervention, and more targeted use of biomaterials, biologics, or cell-based therapies.

In the near term, the most practical molecular application is likely risk stratification to identify individuals who may benefit from targeted prevention. Over time, localized delivery of molecular modulators through biomaterial platforms may offer a more precise way to normalize remodeling and support repair.

## 5 Key challenges and future directions

Despite rapid progress, major biological and translational hurdles still limit the clinical impact of regenerative strategies for pelvic floor disorders. The pelvic floor is a demanding target because implants and biologics must provide early mechanical support, perform under repetitive loading, integrate stably, and avoid chronic inflammation, fibrosis, infection, or mesh-like complications. At the same time, pelvic floor–specific healing biology remains incompletely understood across tissues and life stages, including postpartum and postmenopausal remodeling, which limits rational material design and patient selection.

Methodological heterogeneity is another major barrier. Differences in models, surgical techniques, endpoints, and reporting make studies difficult to compare and obscure what should count as true regeneration beyond short-term histology. The field therefore needs standardized preclinical methods, harmonized functional outcomes, and long-term studies that track symptoms, tissue quality, recurrence, and quality of life.

Regulatory and ethical requirements will also shape translation, especially in preventive or postpartum settings where acceptable risk is low. Combination products, cell-based therapies, and advanced biologics raise additional challenges related to manufacturing consistency, sterility, traceability, long-term safety monitoring, and informed consent requirements.

Future progress will depend on four priorities: better mechanistic understanding of pelvic floor healing, standardized long-term outcome studies, regulatory-ready development pathways, and precision approaches that match therapy to tissue phenotype and risk. Validated molecular, imaging, and biomechanical biomarkers could support early risk stratification and treatment monitoring, while implementation research will be needed to integrate regenerative approaches into real-world pelvic healthcare.

## 6 Conclusion

Regenerative medicine is reshaping pelvic floor care from passive reinforcement toward biologically instructive strategies that aim to restore tissue quality. Advances in degradable biomaterials, bioactive delivery systems, and adjunctive regenerative cues support more constructive remodeling in this mechanically demanding environment.

Its greatest promise may lie in shifting care from treatment alone toward prevention. Because maladaptive remodeling often begins long before overt symptoms or surgery, regenerative interventions may be most effective when used early, particularly in postpartum or other high-risk settings. Durable outcomes will therefore depend on improving healing, not only on correcting anatomy.

Achieving this goal will require pelvic floor–specific mechanistic insight, standardized long-term evaluation, and risk-stratified care pathways that can responsibly bring regenerative therapies into routine practice.
